# 2127. *In vitro* Activities of Ceftaroline and Comparator Agents against Bacterial Pathogens Frequently Causing Community-Acquired Respiratory Tract Infections in Patients from a Global Population: ATLAS Surveillance Program 2018-2021

**DOI:** 10.1093/ofid/ofad500.1750

**Published:** 2023-11-27

**Authors:** Meredith Hackel, Gregory Stone, Daniel F Sahm

**Affiliations:** IHMA, Schaumburg, Illinois; Pfizer, Inc., Groton, Connecticut; IHMA, Schaumburg, Illinois

## Abstract

**Background:**

Community-acquired bacterial pneumonia (CABP) is a frequent cause of patient morbidity and mortality. *Streptococcus pneumoniae*, *Haemophilus influenzae*, and *Moraxella catarrhalis* are frequent etiologic agents of CABP. Ceftaroline fosamil is a parenteral cephem approved treatment of patients with CABP caused by *S. pneumoniae* (including cases with concurrent bacteremia), methicillin-susceptible *Staphylococcus aureus* (MSSA), *H. influenzae*, and some species of Enterobacterales. In this study we report the *in vitro* activity of ceftaroline and comparators against isolates from community-acquired respiratory tract infections (CARTI) collected through a global surveillance program.

**Methods:**

Clinically relevant, non-duplicate, isolates cultured from respiratory specimens by clinical laboratories in 54 countries in 2018-2021 were collected by the ATLAS Surveillance Program central laboratory (IHMA, Schaumburg, IL, USA). Community-acquired infections were defined as those from patients < 48 hours in hospital. In total, 7,886 isolates of *S. pneumoniae*, *H. influenzae*, *M. catarrhalis*, MSSA, and methicillin-resistant *S. aureus* (MRSA) were tested. The isolates (n/percent of total) originated from Asia/South Pacific (1,893/24.0%); Europe (4,283/54.3%); Latin America (671/8.5%); Middle East/Africa (659/8.4%); and North America (Canada only) (380/4.8%). Ceftaroline and comparator agent MICs were determined by CLSI M07 broth microdilution methodology. MICs were interpreted using 2023 CLSI M100 MIC breakpoints.

**Results:**

The *in vitro* activity of ceftaroline and comparator agents is summarized in the table. Greater than 99% of *S. pneumoniae* and 100% of MSSA were susceptible to ceftaroline, including penicillin-nonsusceptible *S. pneumoniae* based on a dosage of 600 mg every 12h. Sixty-two (9.6%) MRSA were ceftaroline-susceptible-dose-dependent (MIC 2-4 µg/mL) based on a dosage of 600 mg every 8h administered over 2h, with the majority from (n) Thailand (9), S. Korea (7), and China (6). Four isolates, from S. Korea (2), China (1), and Ukraine (1) were resistant to CPT (MIC of ≥8 µg/mL). 99.6% of *H. influenzae* were susceptible to ceftaroline.

**Figure:**
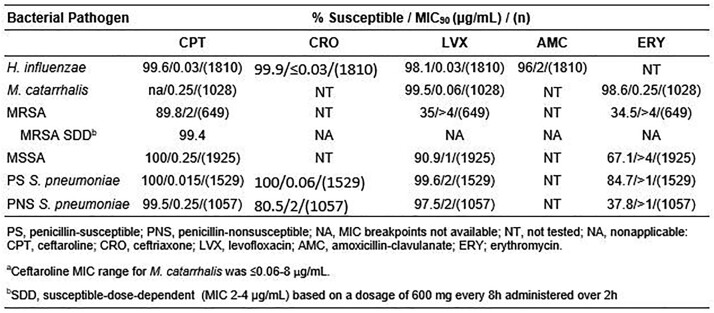

**Conclusion:**

Ceftaroline demonstrated potent *in vitro* activity against current pathogens associated with CABP from a global collection.

**Disclosures:**

**Meredith Hackel, PhD**, Pfizer Inc.: Honoraria|Venatorx: Paid fees for conducting the study and abstract preparation **Gregory Stone, PhD**, Pfizer: Stocks/Bonds **Daniel F. Sahm, PhD**, Merck & Co., Inc.: Honoraria|Pfizer Inc.: Honoraria|Venatorx: Paid fees for conducting the study and abstract preparation

